# Human Embryonic and Fetal Mesenchymal Stem Cells Differentiate toward Three Different Cardiac Lineages in Contrast to Their Adult Counterparts

**DOI:** 10.1371/journal.pone.0024164

**Published:** 2011-09-09

**Authors:** Arti A. Ramkisoensing, Daniël A. Pijnappels, Saïd F. A. Askar, Robert Passier, Jim Swildens, Marie José Goumans, Cindy I. Schutte, Antoine A. F. de Vries, Sicco Scherjon, Christine L. Mummery, Martin J. Schalij, Douwe E. Atsma

**Affiliations:** 1 Department of Cardiology, Leiden University Medical Center, Leiden, The Netherlands; 2 Department of Embryology and Anatomy, Leiden University Medical Center, Leiden, The Netherlands; 3 Department of Molecular Cell Biology, Leiden University Medical Center, Leiden, The Netherlands; 4 Department of Obstetrics and Gynaecology, Leiden University Medical Center, Leiden, The Netherlands; University of Sao Paulo – USP, Brazil

## Abstract

Mesenchymal stem cells (MSCs) show unexplained differences in differentiation potential. In this study, differentiation of human (h) MSCs derived from embryonic, fetal and adult sources toward cardiomyocytes, endothelial and smooth muscle cells was investigated. Labeled hMSCs derived from embryonic stem cells (hESC-MSCs), fetal umbilical cord, bone marrow, amniotic membrane and adult bone marrow and adipose tissue were co-cultured with neonatal rat cardiomyocytes (nrCMCs) or cardiac fibroblasts (nrCFBs) for 10 days, and also cultured under angiogenic conditions. Cardiomyogenesis was assessed by human-specific immunocytological analysis, whole-cell current-clamp recordings, human-specific qRT-PCR and optical mapping. After co-culture with nrCMCs, significantly more hESC-MSCs than fetal hMSCs stained positive for α-actinin, whereas adult hMSCs stained negative. Furthermore, functional cardiomyogenic differentiation, based on action potential recordings, was shown to occur, but not in adult hMSCs. Of all sources, hESC-MSCs expressed most cardiac-specific genes. hESC-MSCs and fetal hMSCs contained significantly higher basal levels of connexin43 than adult hMSCs and co-culture with nrCMCs increased expression. After co-culture with nrCFBs, hESC-MSCs and fetal hMSCs did not express α-actinin and connexin43 expression was decreased. Conduction velocity (CV) in co-cultures of nrCMCs and hESC-MSCs was significantly higher than in co-cultures with fetal or adult hMSCs. In angiogenesis bioassays, only hESC-MSCs and fetal hMSCs were able to form capillary-like structures, which stained for smooth muscle and endothelial cell markers.Human embryonic and fetal MSCs differentiate toward three different cardiac lineages, in contrast to adult MSCs. Cardiomyogenesis is determined by stimuli from the cellular microenvironment, where connexin43 may play an important role.

## Introduction

Despite significant advances in the management of cardiovascular disease, it remains the predominant cause of morbidity and mortality in Western countries [1]. The risk of developing cardiovascular disease increases with age and is associated with progressive impairment of cardiovascular repair mechanisms, including the capacity of the heart to replace damaged cells [2].

In recent years, cell therapy has been studied intensively as novel therapeutic option for cardiac diseases. After transplantation and engraftment of cells in the host myocardium, different mechanisms are thought to be responsible for the improvement in cardiac function, including angiogenesis and cardiomyogenesis [3]. Mesenchymal stem cells (MSCs) are one of the cell types studied in clinical trials for treatment of ischemic heart disease. However, MSCs themselves are prone to the effects of aging and disease and if autologous MSCs are used, may suffer from decreased ability to proliferate, differentiate, home, engraft and exert immunosuppressive effects [4], [5]. Moreover, the developmental stage of tissues and different states of disease alter the microenvironmental regulation of stem cell behaviour [6]. Whether the developmental stage of MSC donor tissue also affects the cardiac differentiation potential of MSCs is not completely understood. While the role of MSCs in cardiac development is largely unknown, several studies indicate that MSCs derived from young cell sources, like umbilical cord blood, appear to retain their primitive characteristics [7], [8]. Also, some of these MSCs seem to possess cardiovascular differentiation potential *in vitro* and *in vivo* [9], [10]. More recently, cells with MSC-like characteristics have been derived from human embryonic stem cells (ESCs) [11]–[15]. These cells resemble hMSCs derived from various tissue sources with respect to morphology, surface marker profile, immunogenicity and differentiation potential toward osteogenic, adipogenic and chrondrogenic lineages [12], [14]. More important, they lack expression of pluripotency-associated markers, and after transplantation, no teratoma formation has been reported [12], [16]. MSCs derived from human ESCs may therefore be mesoderm progenitors like primitive MSCs and may constitute the most primitive committed mesodermal cell type.

In this study, it was investigated whether the developmental stage of the tissue, from which hMSCs were derived, had an effect on the cardiac differentiation potential of these cells. To this end, the ability of hMSCs derived from ESCs, fetal (amniotic membrane, umbilical cord and bone marrow) and adult (adipose tissue and bone marrow) tissue sources to differentiate towards cardiomyocytes, smooth muscle cells and endothelial cells was studied.

## Materials and Methods

### Isolation and culture of neonatal rat cardiomyocytes and fibroblasts

All animal experiments were approved by the Animal Experiments Committee of the Leiden University Medical Center (LUMC) and conform to the Guide for the Care and Use of Laboratory Animals, as stated by the US National Institutes of Health (permit numbers: 09012 and 10236). Neonatal rat ventricular cardiomyocytes (nrCMCs) and fibroblasts (nrCFBs) were isolated and cultured as described previously [10].

### Isolation, culture and characterization of hMSCs

All human-derived tissues were collected based on individual written (parental) informed consent, after approval by the Medical Ethics committee of the LUMC, where all investigations were performed. The investigation conforms with the principles outlined in the Declaration of Helsinki. Human mesenchymal stem cells (hMSCs) were derived from embryonic stem cells (hESC-MSCs), fetal amniotic membrane (amniotic), umbilical cord (UC), bone marrow (BM), adult BM and adipose tissue (adipose). Furthermore, fetal human skin fibroblasts (hSFBs) were also isolated and used as control cells (see online supplement for extensive description of cell isolation and culture procedures). All hMSCs were characterized by flow cytometry, adipogenic and osteogenic differentiation ability, immunocytological analyses, growth kinetics and telomere length. After characterization, cells were labeled with enhanced green fluorescent protein (eGFP) and put in co-culture with nrCMCs or nrCFBs for 10 days to study their cardiomyogenic differentiation potential. Differentiation was assessed by human-specific immunocytological analyses, whole-cell current-clamp recordings, human-specific quantitative reverse transcription-polymerase chain reaction (qRT-PCR) analysis and optical mapping of action potential propagation. In addition, Western Blot analysis was used to study the expression of connexin43 (Cx43) in relation to differentiation of hMSCs. All experiments described below were conducted using hMSCs from passage 3–6.

### Flow cytometry

Analysis of surface marker expression was carried out by flow cytometry using fluorescein isothiocyanate-, phycoerythrin- or allophycocyanin-conjugated antibodies directed against human CD105 (Ancell, Bayport, MN, USA), CD90, CD73, CD45, CD34, CD31, CD24 and stage-specific embryonic antigen-4 (SSEA-4) (all from Becton Dickinson, Franklin Lakes, NJ, USA).

### Adipogenic and osteogenic differentiation

Adipogenesis and osteogenesis of hMSCs were induced by incubating hMSCs in appropriate differentiation media. Lipid accumulation was assessed by Oil Red O (Sigma-Aldrich, St. Louis, MO, USA), while calcium deposits were visualized by staining the cells with 2% Alizarine Red S (Sigma-Aldrich).

### Growth kinetics

Growth kinetics of the hMSCs was analyzed by calculating population doublings (PDs). hMSCs were plated in triplicate and trypsinized every 5 days and subjected to Trypan Blue staining to determine the viable cell concentration using a hemocytometer.

### Relative telomere length

Genomic DNA from experimental and reference samples was obtained using DNAzol (Invitrogen, Breda, The Netherlands). Relative telomere lengths were measured by SYBR Green-based (Qiagen, Valencia, CA, USA) qRT-PCR amplification of telomere repeats (T) and single-copy gene *36B4* (S) in a LightCycler 480 Real-Time PCR System (Roche, Foster City, CA, USA).

### Immunocytochemical analyses

Co-cultures or monocultures were fixed in 4% paraformaldehyde, permeabilized with 0.1% Triton X-100, and stained with primary antibodies. Primary antibodies specific for human lamin A/C (Vector laboratories, Burlingame, CA, USA), SSEA-4, Oct3/4 (both Santa Cruz Biotechnologies, Santa Cruz, CA, USA), Nanog (R&D Systems, Minneapolis, MN, USA), CD90, CD73, and CD105 were used to characterize hESC-MSCs. Co-cultures were stained with α-actinin, Cx43 (both Sigma-Aldrich) and human lamin A/C. Primary antibodies were visualized with Alexa fluor-conjugated antibodies (Invitrogen). Nuclei were stained using Hoechst 33342 (Invitrogen). A fluorescence microscope equipped with a digital camera (Nikon Eclipse, Nikon Europe, Badhoevedorp, The Netherlands) and dedicated software (Image-Pro Plus, Version 4.1.0.0, Media Cybernetics, Silver Spring, MD, USA) were used to analyze data. All cultures were treated equally using the same antibody dilutions and exposure times.

### Electrophysiological measurements in pharmacologically uncoupled hMSCs in co-culture with nrCMCs

In order to study functional cardiomyogenic differentiation in hMSCs, either eGFP-labeled fetal or adult hMSCs were put in co-culture with nrCMCs and studied as described previously [10]. In brief, at day 10 of co-culture, 180 µmol/L of 2-aminoethoxydiphenyl borate (2-APB) (Tocris, Ballwin, MO, USA) was added to the extracellular solution, resulting in gap junction uncoupling [17], [18], which allowed for single-cell studies within the co-culture. Next, whole-cell current-clamp recordings were performed in eGFP-labeled hMSCs.

### Human-specific quantitative reverse transcription PCR

Total cellular RNA was extracted from monocultures of hMSCs and from co-cultures consisting of hMSCs and nrCMCs using the RNeasy Mini kit (Qiagen). Oligo (dT)-primed reverse transcription was performed on 2 µg of total cellular RNA and the resultant cDNA was used for PCR amplification using SYBR Green. To detect changes in cardiac and pluripotency gene expression levels, only human-specific primers were used. The expression of the genes of interest was normalized to that of the housekeeping gene *glyceraldehyde-3-phosphate dehydrogenase* (GAPDH). Specific primer information and annealing temperatures are provided in the online supplement. Data were analyzed using the ΔCt method.

### Optical mapping to determine conduction velocity in co-cultures of nrCMCs and different types of hMSCs

Action potential conduction velocity (CV) was investigated on a whole-culture scale in wells of a 24-well plate by optically mapping using the voltage-sensitive dye di-4-ANEPPS (Invitrogen). The measurements were performed 10 days after seeding of either 8×10^5^ nrCMCs (nrCMC monoculture) or 8×10^5^ nrCMCs plus 8×10^4^ nrCFBs or 8×10^4^ hMSCs (nrCMC/nrCFB or nrCMC/hMCS co-cultures) per well. The co-cultures were mapped using the Ultima-L optical mapping setup (SciMedia, Costa Mesa, CA, USA). Optical signal recordings were analyzed using Brain Vision Analyze 0909 (Brainvision Inc, Tokyo, Japan). The CV of all (co-)cultures was determined in a blinded manner.

### Assessment of angiogenesis

All types of hMSCs were were plated on Matrigel (Becton Dickinson) and cultured in Endothelial Growth Medium-2 (Cambrex IEP, Wiesbaden, Germany) containing 100 ng/mL recombinant human VEGF-A_165_ (R&D Systems) up to 24 h to determine their ability to form capillary-like structures. Following culture under angiogenic conditions, cells were fixed and stained with antibodies specific for smooth muscle myosin heavy chain (smMHC; Sigma-Aldrich) and platelet/endothelial cell adhesion molecule-1 (PECAM-1; Santa Cruz).

### Western Blot analysis

Homogenates were made from at least 5 different isolations of hMSCs per source. Equal amounts of protein were size-fractionated in a 12% NuPage Tris-Acetate gel (Invitrogen) and transferred to a Hybond-P PVDF membrane (GE Healthcare, Waukesha, WI, USA). This membrane was incubated for 1 h with an antibody against Cx43 followed by incubation with horse radish peroxidase conjugated goat anti-rabbit secondary antibody (Santa Cruz). To check for equal protein loading, the housekeeping protein GAPDH (Chemicon International, Temecula, CA, USA) was used.

### Statistics

Experimental results were expressed as mean±standard deviation (SD) for a given number (n) of observations. Data was analyzed by Student's t-test for direct comparisons. Analysis of variance followed by the appropriate post-hoc analysis was performed for multiple comparisons. Statistical analysis was performed using SPSS 16.0 for Windows (SPSS Inc, Chicago, IL, USA). Differences were considered statistically significant at *P*<0.05.

A detailed description of the materials and methods can be found in the Supplemental Materials and Methods S1.

## Results

### Isolation and characterization of hMSCs

All types of hMSCs displayed a spindle-shaped morphology (Figure S1 A1–F1). To evaluate MSC properties, their surface phenotype and adipogenic and osteogenic differentiation capacity were studied. All types of hMSCs were negative for CD31 (endothelial cell marker), CD34, CD45 (hematopoietic cell markers), and SSEA-4 (embryonic stem cell marker), whereas they were positive for CD90, CD73 and CD105 (mesenchymal cell markers). Furthermore, hESC-MSCs were negative for CD24, indicating absence of hESCs in our cultures (Table S1). *In vitro* differentiation assays confirmed that all types of hMSCs were able to differentiate into adipocytes and osteoblasts thus confirming their multipotent differentiation potential (Figure S1 A2–F2 and A3–F3, respectively).

### Growth kinetics

Comparison of the growth kinetics of hESC-MSCs with those of fetal or adult hMSCs showed that hESC-MSCs had a significantly larger replication capacity during 20 days in culture (35.1 PDs) than any of the fetal hMSCs types (22.3–31.6 PDs) and both types of adult MSCs (6.4–12.8 PDs) (*P*<0.001). Fetal hMSCs also proliferated more rapidly and grew to higher densities than both types of adult hMSCs (*P*<0.001) (Figure S1 G).

### Telomere length

Replicative stability of hMSCs was determined by estimating their relative telomere lengths (i.e. telomere repeat copy number to single gene copy number [T/S] ratio). Relative telomere length was significantly longer in hESC-MSCs (4.87±0.7) and in fetal hMSCs (2.48±0.4) than in adult hMSCs (0.89±0.2) (*P*<0.05; n = 10 samples from different isolations for each hMSC group). Relative telomere length was also significantly longer in hESC-MSCs than in fetal hMSCs (*P*<0.05; n = 10 samples from different isolations for each hMSC group) (Figure S1 H).

### Immunocytological characterization of hESC-MSCs

**Figure 1:**
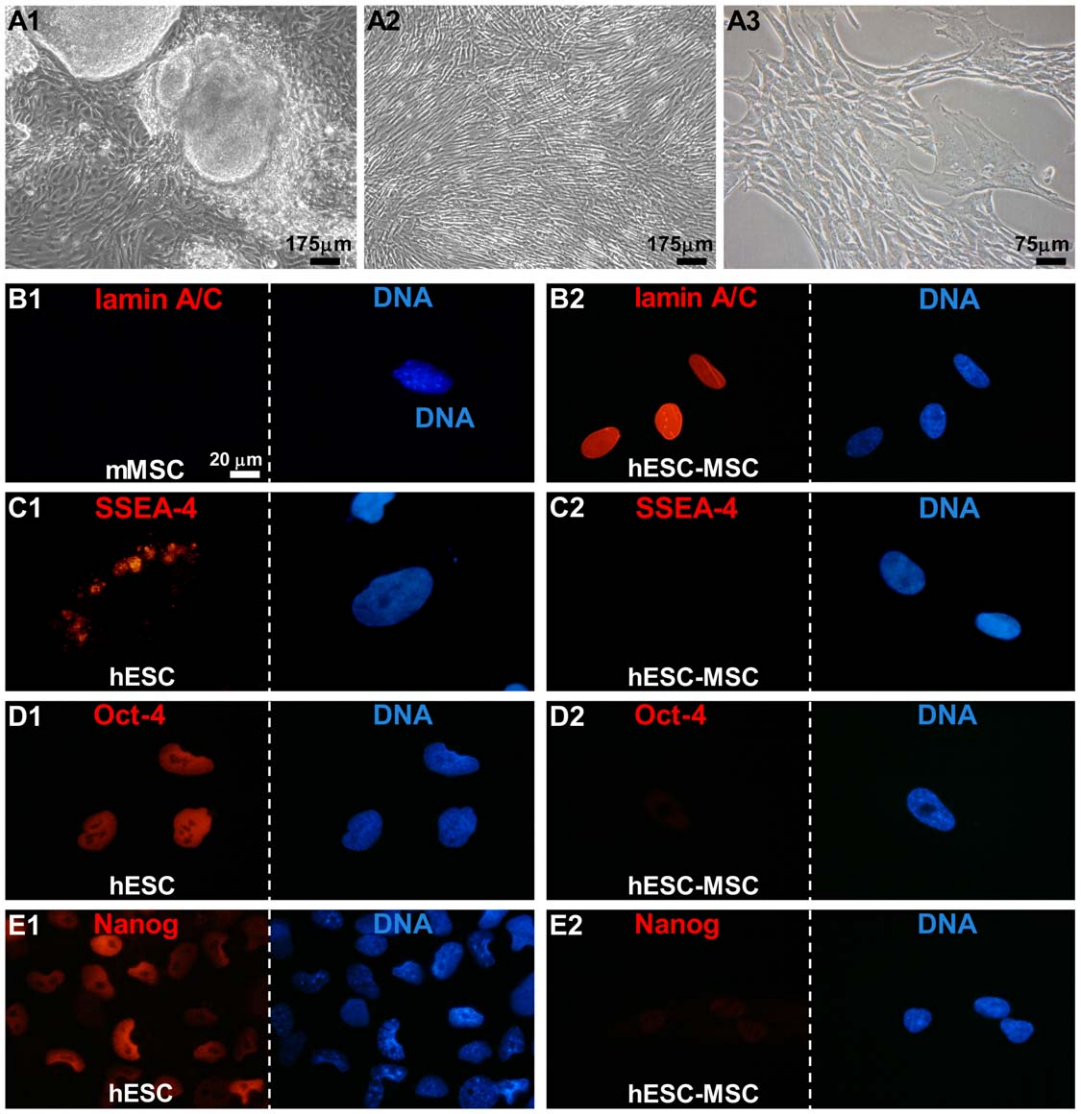
Characterization of hESC-MSCs. (A1) Bright field image of a hESC colony in which the cells at the periphery are differentiating toward spindle-shaped fibroblast-like cells and (A2–A3) pure cultures of hESC-MSC. (B1–B2) Confirmation of the human origin of the hMSCs derived from hESC colonies with the aid of a human-specific lamin A/C antibody. Incubation of murine MSCs (mMSCs; negative control cells) with this antibody (B1) did not produce signal corroborating its species specificity. (C1–E2) Immunostaining of hESC colonies and hMSCs derived from these colonies for the embryonic stem cell marker SSEA-4 and the pluripotency markers Oct-4 and Nanog. Nuclei were detected with Hoechst.

All fibroblast-like cells derived from the hESC colonies (Figure 1A1–A3) were recognized by a monoclonal antibody specific for human lamin A/C confirming the human origin of these cells (Figure 1B2). Murine MSCs (mMSCs), which served as a negative control, were negative for this marker (Figure 1B1). In addition, hESC-MSCs were negative for the undifferentiated hESC marker SSEA-4 and the pluripotency markers Oct-4 and Nanog (Figure 1C2–E2) in contrast to the hESC colonies from which the fibroblast-like cells were derived (Figure 1C1–E1). However, hESC-MSCs were positive for the mesenchymal cell markers CD90, CD73 and CD105 (Figure S2 A1–A3).

### Assessment of cardiomyogenic differentiation

#### Human-specific immunocytological evaluation

**Figure 2:**
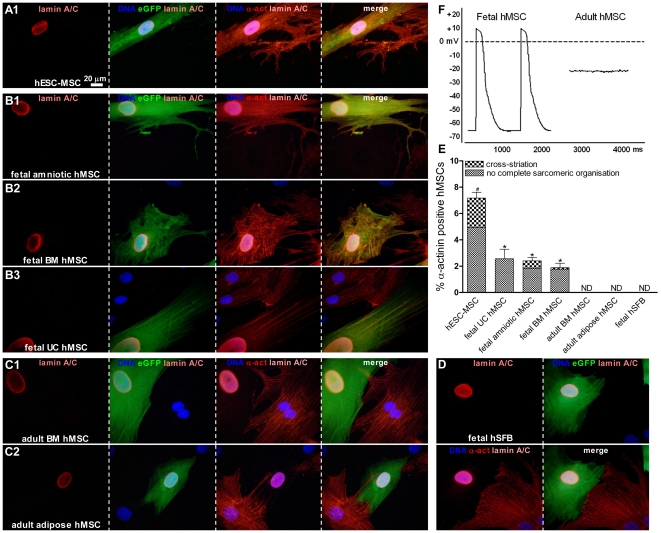
Immunocytological assessment of cardiomyogenic differentiation of different types of hMSCs after 10 days of co-culture with nrCMCs. (A1–B3) A fraction of eGFP-labeled, human-specific lamin A/C positive hESC-MSCs and fetal amniotic, BM and UC hMSCs expressed α-actinin (indicated as α-act), while (C1–C2) adult BM and adipose hMSCs did not. (D) eGFP-labeled human fetal skin fibroblasts (hSFBs; negative control cells) in co-culture with nrCMCs did not stain positive for α-actinin. (E) Quantitative analysis of the cardiomyogenic differentiation of different types of hMSCs. The graph is based on a minimum of 1,200 cells analyzed from 4 separate isolations per hMSC type. ^#^ *P*<0.001 vs all fetal and adult hMSC types; ^*^ *P*<0.05 vs adult hMSCs; ND is not detected. (F) Intracellular electrophysiological measurements in fetal (amniotic) and adult (adipose) hMSCs at day 10 of co-culture with nrCMCs and after pharmacological uncoupling of gap junctions. Intrinsic action potentials could be recorded from eGFP-labeled fetal cells, while adult hMSCs showed only steady membrane potentials.

At day 10 of co-culture with nrCMCs, 7.17±0.4% eGFP-labeled hESC-MSCs (n = 1,500 cells analyzed from 5 different isolations) were positive for the sarcomeric protein α-actinin (Figure 2A1 and 2E), which was a significantly higher fraction than the percentage of fetal hMSCs staining for α-actinin (fetal amniotic MSCs 2.15±0.2%, fetal BM MSCs 1.8±0.3% and fetal UC MSCs 2.56±0.7%, n = 1,200 cells analyzed from 4 different isolations per type of fetal hMSC) (*P*<0.001) (Figure 2B and 2E). Furthermore, in some of the hESC-MSCs (30.8%), fetal amniotic MSCs (25.8%) and fetal BM MSCs (5.89%) α-actinin was distributed in a cross-striated pattern typical for CMCs. After 10 days of co-culture with nrCMCs, adult BM and adipose MSCs did not stain for α-actinin (n = 1,200 cells analyzed from 4 different isolations per type of adult hMSC) (Figure 2C and 2E). eGFP-labeled fetal hSFBs in co-culture with nrCMCs were not positive for α-actinin (n = 1,200 cells analyzed from 4 different isolations) (Figure 2D and 2E) indicating that not all fibroblastic human cell types acquire cardiomyocyte properties in co-culture with nrCMCs. To exclude fusion of nrCMCs with hMSCs or secondary transduction of nrCMCs with eGFP, all co-cultures were also stained for human-specific lamin A/C. None of the eGFP positive cells were negative for human-specific lamin A/C or contained multiple nuclei (n≥8,500 eGFP-positive cells analyzed) confirming the validity of the assay system.

**Figure 3:**
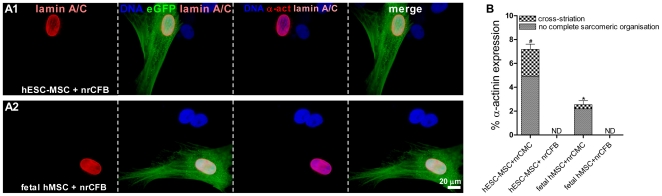
Study of cardiomyogenic differentiation in hESC-MSCs and fetal hMSCs after co-culture with nrCFBs for 10 days assessed by immunocytological analysis. (A1–A2) No α-actinin expression, indicated as α-act, was detected in eGFP-labeled, human-specific lamin A/C positive hESC-MSCs or fetal hMSCs after co-incubation with nrCFBs. Nuclei were detected with Hoechst. (B) Quantitative analysis of cardiomyogenic differentiation of hESC-MSCs and all of the fetal hMSC types co-cultured with nrCFBs or nrCMCs. The graph is based on a minimum of 1,200 cells analyzed from 3 separate isolations per hMSC type. ^#^ *P*<0.001 vs hESC-MSCs and fetal hMSCs co-cultured with nrCFBs and fetal hMSCs in co-culture with nrCMCs; ^*^ *P*<0.01 vs fetal hESC-MSCs and fetal hMSCs co-cultured with nrCFBs; ND is not detected.

To assess the influence of the cellular microenvironment on cardiomyogenic differentiation of hESC-MSCs and fetal hMSCs the presence of α-actinin in these cells after 10 days of co-culture with nrCFBs rather than nrCMCs was determined. Alpha-actinin was not expressed by hESC-MSCs (Figure 3A1) or any of the fetal hMSC types (Figure 3A2) after co-culture with nrCFBs (n = 1,200 cells analyzed from 3 different isolation of each hMSC type) (Figure 3B).

#### Intracellular electrophysiological measurements in pharmacologically uncoupled hMSCs in co-culture with nrCMCs

Patch-clamp recordings were obtained from eGFP-labeled fetal and adult hMSCs at day 10 of co-culture with nrCMCs after electrical isolation through incubation with the gap junction uncoupler 2-APB. Action potentials could only be measured in fetal hMSCs (n = 5, amniotic), this in contrast to adult hMSCs (n = 8, adipose), which only showed steady membrane potentials (Figure 2F). Selection of cells was based on eGFP-labeling and the presence of 2–4 nrCMCs adjacent to the cells of interest.

#### Human-specific qRT-PCR analysis to detect pluripotency and cardiac differentiation

**Figure 4:**
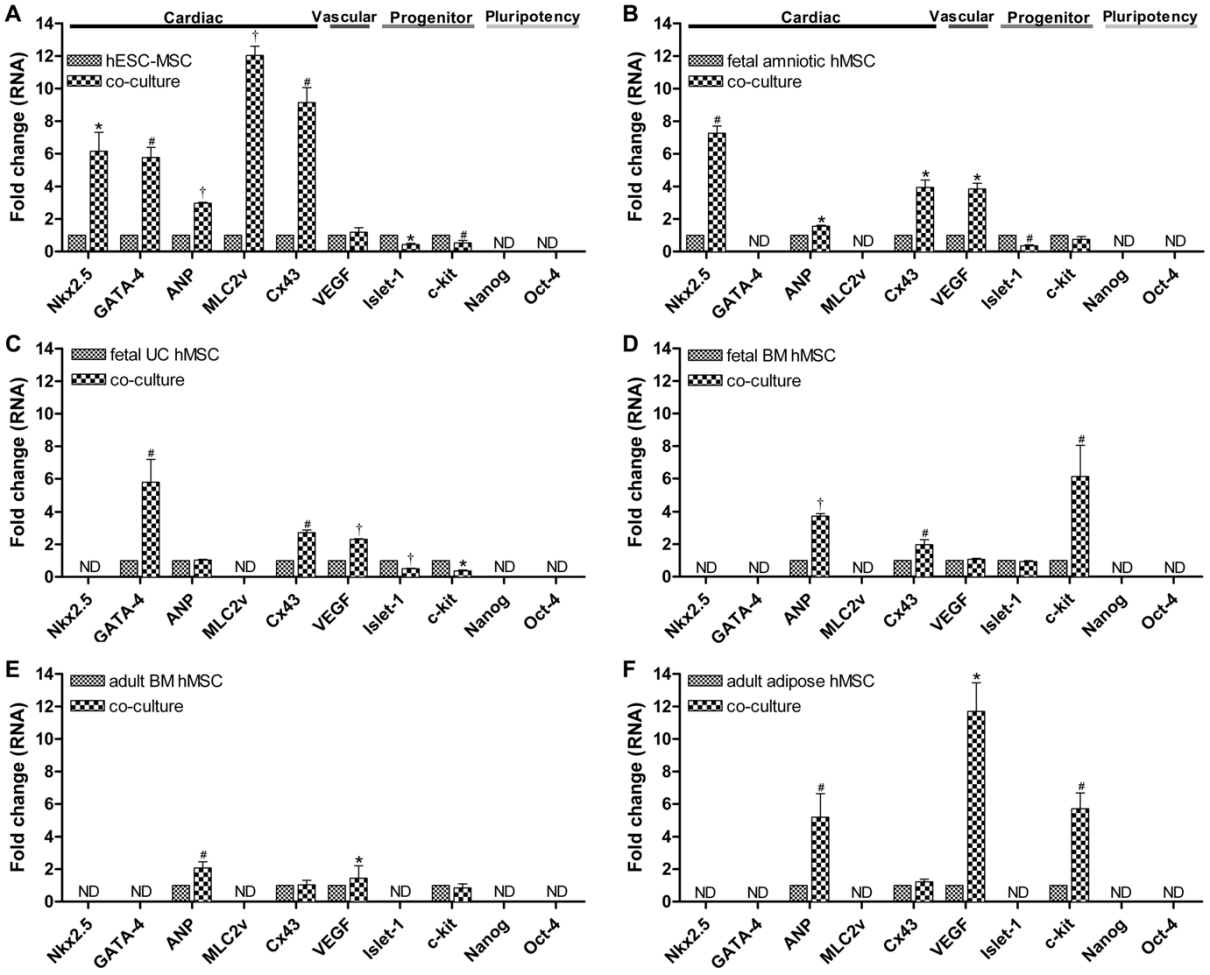
Analysis by qRT-PCR of expression of pluripotency and cardiac genes in hMSCs alone or after co-incubation with nrCMCs. (A) hESC-MSCs expressed most cardiac-specific genes, which were significantly upregulated after co-incubation with nrCMCs. Expression of the cardiac progenitor genes, *Islet-1* and *c-kit*, was downregulated in the presence of nrCMCs. (B–D) The fetal hMSC types expressed a variety of cardiac-specific genes, which were upregulated after co-culture with nrCMCs. *Islet-1* and *c-kit* mRNA levels decreased in the presence of nrCMCs with the exception of the upregulation of *c-kit* gene expression in fetal BM MSCs following their co-culture with nrCMCs. (E–F) *ANP*, *Cx43*, *VEGF* and *c-kit* gene expression was detected in adult hMSCs before and after co-incubation with nrCMCs. (A–F) hMSCs did not express the pluripotency genes Oct-4 and Nanog. ^#^ *P*<0.05 vs specific hMSC monoculture; ^*^ *P*<0.01 vs specific hMSC monoculture; ^†^ *P*<0.001 vs specific hMSC monoculture.

qRT-PCR showed that at the mRNA level, hESC-MSCs expressed the following cardiac markers: Nkx2.5, GATA-4, ANP, MLC2v and Cx43. These mRNAs were upregulated after co-culture with nrCMCs (P<0.001 for both ANP and MLC2v; P<0.01 for Nkx2.5; P<0.05 for both GATA-4 and Cx43). However, Islet-1 and c-kit mRNA levels were lower in co-cultured hESC-MSCs compared to hESC-MSC monocultures (P<0.01 and P<0.05, respectively). No significant difference in gene expression of VEGF was detected in hESC-MSCs before or after co-incubation with nrCMCs (Figure 4A). Fetal amniotic hMSCs showed an increase in Nkx2.5 (P<0.05), ANP, Cx43 and VEGF (all P<0.01) gene expression after co-culture with nrCMCs, while Islet-1 mRNA levels were decreased under these circumstances (P<0.05). No difference in c-kit gene expression was detected in these fetal hMSCs before or after co-incubation with nrCMCs (Figure 4B). In fetal UC hMSCs, mRNA levels of GATA-4, Cx43 (both P<0.05) and VEGF (P<0.001) were significantly upregulated after their co-culture with nrCMCs, while Islet-1 and c-kit gene expression were downregulated (P<0.001 and P<0.01, respectively). No change in the expression of ANP was detected in fetal UC hMSCs following co-incubation with nrCMCs (Figure 4C). Fetal BM hMSCs showed an increase in ANP (P<0.001), Cx43 and c-kit (both P<0.05) gene expression co-incubation with nrCMCs, while no difference in Islet-1 and VEGF mRNA levels were detected under these circumstances (Figure 4D). At the mRNA level, ANP, Cx43, VEGF and c-kit were detected in adult BM and adipose hMSCs. However, after co-culture with nrCMCs, only the expression of ANP (P<0.05) and VEGF (P<0.01) increased in both types of adult hMSCs. In adult adipose hMSCs c-kit mRNA levels were also upregulated in the presence of nrCMCs (P<0.05) (Figure 4E–4F). Oct-4, Nanog, cTnI and β-MHC gene expression was not detected in any of the hMSC types. qRT-PCR analysis of RNA from appropriate human control samples confirmed the functionality of all human-specific primer pairs. The same primer pairs did not give rise to amplification products using nrCMC RNA as starting material. Expression of the qRT-PCR target genes in nrCMCs was confirmed using rat-specific primers. Quantitative differences in gene expression between hMSCs cultured alone or with nrCMCs are given in Table S2.

#### Optical mapping analysis to determine action potential CV in co-cultures of nrCMCs with different types of hMSCs

**Figure 5:**
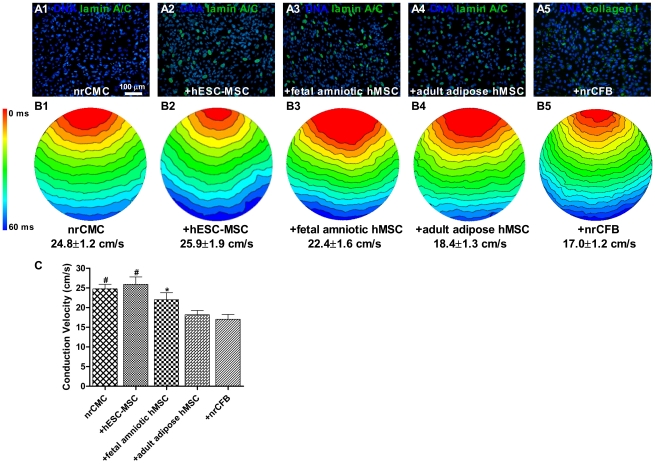
Assessment of CV by optical mapping in co-cultures of nrCMCs and different types of hMSCs. (A) The presence of hMSCs and nrCFBs after optical mapping was confirmed by immunostaining for human-specific lamin A/C and collagen type I, respectively. Nuclei were detected with Hoechst. (B) Activation maps of the different (co-)cultures reveal significantly higher CVs in nrCMCs monocultures and in hESC-MSCs/nrCMC and fetal amniotic hMSCs/nrCMC co-cultures than in co-cultures of nrCMCs with adult adipose hMSCs or with nrCFBs. CVs were also significantly higher in nrCMC monocultures and in hESC-MSCs/nrCMC co-cultures than in co-cultures between nrCMCs and fetal amniotic hMSCs. Spacing of isochronal lines in activation maps is 4 ms, and colors indicate temporal sequence of activation, starting from the red area. (C) Bar graph of the CVs in nrCMC monocultures and in co-cultures (+) between nrCMCs and nrCFBs or different types of hMSCs as indicated. ^#^ *P*<0.001 vs fetal amniotic hMSCs, adult adipose hMSCs and nrCFBs co-incubated with nrCMCs; ^*^ *P*<0.01 vs adult adipose hMSCs and nrCFBs co-incubated with nrCMCs.

CV in nrCMC co-cultures with hESC-MSCs (25.9±0.9 cm/s) was similar to the CV in nrCMC cultures alone (24.8±1.2 cm/s) (Figure 5B1–B2 and 5C). However, it was significantly higher than in co-cultures of nrCMCs with fetal amniotic hMSCs (22.0±1.8 cm/s), adult adipose hMSCs (18.2±1.1 cm/s) and nrCFBs (17.0±1.2 cm/s) (*P*<0.001; n≥15 co-cultures with each cell type) (Figure 5B3–B5 and C). CV was also higher in co-cultures of nrCMCs with fetal amniotic hMSCs than in co-cultures with adult adipose hMSCs or with nrCFBs (*P*<0.001) (Figure 5B3–B5 and C).

### *In vitro* angiogenesis assays

**Figure 6:**
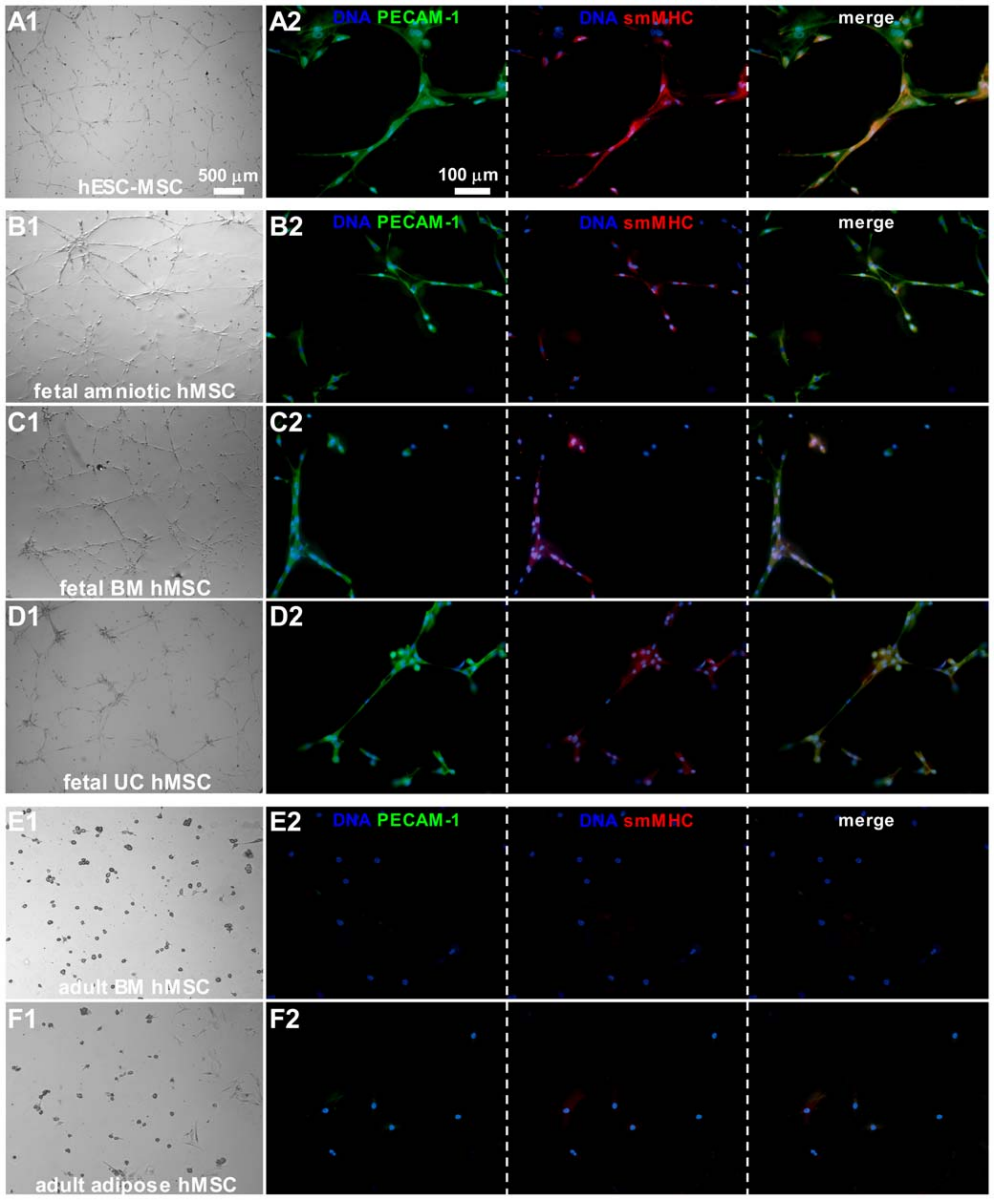
Angiogenic differentiation capacity of different types of hMSCs assessed by formation of capillary-like structures and expression of angiogenic markers following their culture on Matrigel. (A1–D1) Bright field images show that hESC-MSCs and fetal hMSCs were able to form stable cellular networks on a basement membrane matrix, while (E1–F1) adult hMSCs could not. (A2–D2) hESC-MSCs and all fetal hMSC types stained positive for the endothelial cell protein, PECAM-1, and the smooth muscle cell protein, smMHC, while (E2–F2) the adult hMSCs were negative for these markers. Nuclei were stained with Hoechst.

hESC-MSCs and all types of fetal hMSCs were able to form capillary-like structures on Matrigel (n≥5 isolations of each hMSC type incubated in triplicate) (Figure 6A1–D1). These networks stained positively for the smooth muscle marker smMHC and the endothelial cell marker PECAM-1 (Figure 6A2–D2). Formation of cellular networks was established by hESC-MSCs 12 h after incubation on Matrigel. For hMSCs derived from the fetal sources it took 18 h to establish capillary-like networks. Adult BM and adipose tissue hMSCs failed to form capillary-like structures (n≥5 isolations of each hMSC type incubated in triplicate) (Figure 6E–F). Formation of vessel-like networks was checked every hour for a total period of 24 h.

### Evaluation of Cx43 expression levels

**Figure 7:**
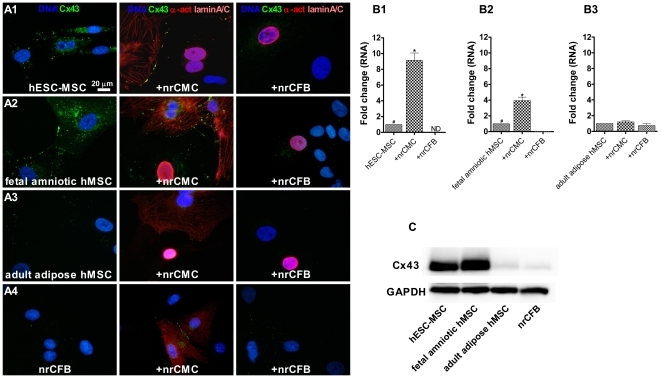
Analysis by immunofluorescence microscopy, qRT-PCR and Western blotting of *Cx43* expression in hMSC monocultures and in co-cultures of nrCMCs or nrCFBs with different types of hMSCs. (A1–A2) Immunocytological analysis shows high Cx43 levels in monocultures of hESC-MSCs and fetal amniotic hMSCs. Cx43 was also detected at the interfaces of these young hMSCs with nrCMCs but not with nrCFBs. nrCMCs were visualized by staining with α-actinin (indicated as α-act), while an antibody against human-specific lamin A/C was used to detect hMSCs. Nuclei were detected with Hoechst. (A3–A4) Adult adipose hMSCs contain very low amounts of Cx43 in both monocultures and co-cultures with nrCMCs or nrCFBs. Also in nrCFB monocultures and nrCFB/nrCMC co-cultures Cx43 is barely detectable. (B1–B3) Bar graphs of the assessment by qRT-PCR of *Cx43* mRNA levels in hESC-MSC, fetal amniotic hMSC and adult adipose hMSC monocultures and co-cultures (+) of these cells with either nrCMCs or nrCFBs as indicated. (C) Picture of representative part of a Western blot showing that hESC-MSCs and fetal amniotic hMSCs contain large amounts of Cx43 in contrast to adult adipose hMSCs and nrCFBs. The housekeeping protein glyceraldehyde-3-phosphate dehydrogenase (GAPDH) was used to check for equal protein loading. ^#^ *P*<0.05 vs hMSCs in co-culture with nrCFBs; ^*^ *P*<0.01 vs hMSC monocultures and hMSCs in co-culture with nrCFBs.

To further analyze mechanisms underlying differences in cardiomyogenic potential, Cx43 expression levels were evaluated. Immunocytological analysis showed that Cx43 protein expression was more abundant in hESC-MSCs and fetal amniotic MSCs than in adult adipose MSCs and nrCFBs (n = 5 isolations of each hMSC type were assessed) (Figure 7A). These results were confirmed by Western blot analysis (n = 5 different isolations for each cell type) (Figure 7C). Cx43 expression was also detected at the interfaces between nrCMCs and hESC-MSCs or fetal MSCs, while under equivalent staining conditions it was not present at contact-areas between nrCMCs and adult hMSCs or nrCFBs (Figure 7A). Moreover, Cx43 did not line borders between nrCFBs and any of the types of hMSCs (n≥1,500 cells analyzed from 5 isolations of each cell type under each condition) (Figure 7A). qRT-PCR showed a significant increase in *Cx43* expression following the incubation of hESC-MSCs or fetal amniotic hMSCs with nrCMCs (9.14±0.9 (*P*<0.01) and 3.94±0.5 fold (*P*<0.001), respectively), while no (hESC-MSCs) or 19.4±0.1 fold less (fetal amniotic hMSCs) *Cx43* mRNA was detected in co-cultures with nrCFBs (*P*<0.05) (Figure 7B1–B2). No significant difference in *Cx43* mRNA levels was detected between adult adipose tissue hMSCs cultured alone or together with nrCMCs or nrCFBs (Figure 7B3).

## Discussion

Key findings of the present study are: 1) Differentiation potential of hMSCs toward three cardiac cell lineages depends on the developmental stage of donor tissue; 2) Cardiomyogenesis of hMSCs is influenced by stimuli from the cellular microenvironment; and 3) The propensity of different types of hMSCs to acquire properties of heart muscle cells correlates with Cx43 expression levels.

### Development and Stemness

During embryonic development, stem cells contribute to organ formation, while later in life these cells or their derivatives are involved in repair and regeneration of organs [6]. However, with increasing age, the potential of stem cells declines [19], [20]. This decrease in the regenerative ability is associated with cumulative organ dysfunction, which may lead to increased morbidity and mortality. Consistent with this developmental stage-dependent decline in function of stem cells, this study showed that MSCs derived from hESCs have significantly greater proliferative capacity and longer telomeres than MSCs derived from human fetal tissue, or adult hMSCs. The intrinsic age-associated decrease in telomere length is one of the mechanisms that contributes to the loss of stem cell properties with age [21]. The results of this first-time direct comparison of the proliferative capacity of hESC-MSCs, fetal hMSCs and adult hMSCs are in line with previous studies [12], [13], [22]–[24]. All together, these findings show that MSCs derived from human ESCs are more immature and display greater stemness than those derived from fetal tissues.

### MSCs and cardiac differentiation

In the present study, it was also shown that hMSCs derived from either embryonic or fetal sources have the capacity to undergo cardiac differentiation, while those derived from adult sources do not. Our results revealed that developmental stage of the donor tissue not only influences the ability of hMSCs to differentiate into CMCs but also their capacity to undergo smooth muscle and endothelial differentiation. This information may be of value in extending the repertoire of cells considered suitable for studies of cardiac repair. We showed that after co-culture with nrCMCs the sarcomeric protein α-actinin is expressed by a significantly higher percentage of hESC-MSCs than of fetal hMSCs, while it was not expressed by adult hMSCs. Although the cardiomyogenic potential of MSCs derived from neonatal sources has been described before, a direct comparison of hMSCs derived from embryonic, fetal and adult sources was not conducted [9], [10]. Controversy still exists on whether MSCs actually differentiate into CMCs or whether this apparent cardiomyogenesis is due to fusion of MSCs with CMCs [25]. Therefore, in all our co-culture experiments eGFP-transduced hMSCs were stained for human-specific lamin A/C. Neither multinucleated eGFP positive cells nor eGFP positive cells negative for human-specific lamin A/C were detected. In addition, only human-specific primers were used in the qRT-PCR experiments to exclude detection of rat cardiac-specific genes expressed by nrCMCs. Accordingly, the cardiomyogenic differentiation of hMSCs observed in our experiments does not result from cell fusion. Importantly, the records of intrinsic action potentials in eGFP positive cells at day 10 of co-culture provides direct evidence for functional cardiomyogenic differentiation.

With respect to smooth muscle and endothelial differentiation, only hESC-MSCs and fetal hMSCs were able to form capillary-like structures on Matrigel. These networks stained positive for the smooth muscle marker smMHC and the endothelial marker PECAM-1. Previous studies showed that adult BM-derived MSCs were able to form capillary-like structures after priming the cells in endothelial differentiation medium. However, after priming, UC hMSCs had higher endothelial potential than adult BM hMSCs [26], [27].

Concerning the underlying mechanisms why adult hMSCs do not form CMCs, Cx43 expression may be of importance. In this study, hMSCs are co-cultured with nrCMCs, resulting in physical contact between these two cell types, and allowing the possibility for gap junction formation. Gap junctions, which in the ventricles are mainly formed by Cx43, allow a low-resistant spread of chemical and electrical signals between adjacent cells [28]. Interestingly, hMSCs derived from adult human sources express very low levels of Cx43, both at mRNA and protein level, as compared to hMSCs derived from embryonic and fetal sources. In addition, hMSCs, which underwent functional cardiomyogenic differentiation, were always adjacent to native CMCs, naturally containing high levels of Cx43. As hMSCs are able to form functional gap junctions with adjacent CMCs [29], electrical and chemical interaction can occur between both cell types and this was shown to play a role in cardiomyogenic differentiation [30]. Interestingly, co-culture of MSCs with cardiac fibroblasts, expressing very low levels of Cx43, did not result in cardiomyogenic differentiation. In fact, using human-specific primers, *Cx43* expression levels in hMSCs were shown to decrease significantly in this microenvironment.

As Cx43 plays an essential role in myocardial electrical conduction across the ventricular muscle, and cardiomyogenic differentiation would make non-excitable MSCs become excitable, we also studied the CV across co-cultures of hMSCs with nrCMCs. It was shown that co-cultures with hESC-MSCs showed a significantly higher CV than those with fetal hMSCs and adult hMSCs, while the CV in co-cultures with fetal hMSCs was also significantly higher than that in those with adult hMSCs. Of note, embryonic MSCs did not only show high expression of Cx43, but had the greatest cardiomyogenic differentiation potential. The type of MSC that contributes to the highest CV may be preferable to accomplish functional integration with host myocardium after transplantation.

### MSC transplantation for cardiac diseases

Besides providing new insights into the factors that stimulate cardiac differentiation, the findings of this study may also have implications for the use of MSCs in patients. Currently, in myocardial cell therapy studies mainly autologous cells from aged patients suffering from chronic diseases are used. Based on our results, it may be expected that the therapeutic effects of MSC transplantation, to improve cardiac function, are affected by intrinsic properties of the transplanted cells. Furthermore, the beneficial effects of stem cell therapy in the damaged heart seems to be largely mediated by paracrine factors promoting neo-angiogenesis and CMC survival with little evidence of the differentiation of transplanted cells into CMCs [3]. As these effects will be of limited help in case of extensive loss of myocardial tissue there is still a great demand for stem cells that can differentiate in vivo into new CMCs. In this study, we have shown that hMSCs of prenatal origin can differentiate into functional CMCs and that this process is dependent on instructive cues provided by neighboring CMCs. Therefore selection of MSCs from donor sources, such as the umbilical cord or amniotic membrane/fluid, may be an attractive source of immature or young cells for autologous cell transplantation. Even allogeneic transplantation may be considered as MSCs are reported to have immunomodulatory properties [31]–[33]. However, many aspects related to transplantation of cardiomyogenic stem cells need to be studied in more detail before optimal therapeutic efficacy and minimal hazardous potential can be achieved.

### Conclusions

Human MSCs of embryonic stem cell or fetal but not adult origin can differentiate into three cardiac lineages: cardiomyocytes, endothelial cells and smooth muscle cells. The ability to undergo functional cardiomyogenic differentiation is amongst others determined by the microenvironment of the cells, in particular their communication with adjacent cell types. The gap junction protein Cx43 may play an important role in this differentiation process.

### Study limitations

It would have been more clinically relevant to co-culture the different hMSC subtypes with adult human hCMCs, but obtaining these cells in the numbers needed to conduct these experiments seems not feasible. Furthermore, adult hCMCs cannot be cultured long enough to perform some of the key experiments described in this paper.

## Supporting Information

Methods S1 A detailed description of the materials and methods can be found in this supporting information file.
(DOC)

Figure S1 Cellular characteristics of hMSCs. (A1–F1) Bright field images of cultured hMSCs displaying a spindle-shaped morphology. (A2–F2) Presence of oil red O-stained fat vacuoles after adipogenic differentiation. (A3–F3) Calcium depositions after osteogenic differentiation was visualized by alizarine red S staining. (A) hESC-MSC; (B) fetal amniotic hMSC; (C) fetal UC hMSC; (D) fetal BM hMSC; (E) adult BM hMSC; (F) adult adipose hMSC. (G) Growth kinetics of the different types of hMSCs estimated by cumulative population doublings over 20 days (^*^ *P*<0.001 vs fetal hMSCs and adult hMSCs, ^#^ *P*<0.001 vs adult hMSCs). (H) Mean relative telomere lengths of hESC-MSCs, all fetal hMSC types and both adult hMSC types (^*^ *P*<0.05 vs fetal hMSCs and adult hMSCs, ^#^ *P*<0.05 vs adult hMSCs).
(TIF)

Figure S2 Immunocytological characterization of hESC-MSCs for MSC surface markers. Immunostaining of hESC-MSCs for CD90, CD105 and CD73 (A1–A3) showed that these cells were positive for these established MSC surface markers.
(TIF)

Table S1 Analysis of surface marker expression. All hMSC types were positive for the established MSC surface markers CD105, CD90 and CD73. They were negative for the hematopoietic, endothelial and embryonic stem cell markers CD45 and CD34, CD31 and SSEA-4, respectively. The hESC-MSCs were also negative for CD24, a protein present on the surface of hESCs. Mean percentages ± standard deviations are given; n = 6 for each group. NT is not tested.
(DOC)

Table S2 qRT-PCR analysis to detect mRNAs associated with cardiac differentiation. Indicated is the fold change in the expression of cardiac genes in hMSCs cultured alone or together with nrCMCs. ^#^ *P*<0.05 vs hMSC monoculture; ^*^ *P*<0.01 vs hMSC monoculture; †*P*<0.001 vs hMSC monoculture; ND is not detected.
(DOC)
