# Supplementary material for: Human Embryonic and Fetal Mesenchymal Stem Cells Differentiate toward Three Different Cardiac Lineages in Contrast to Their Adult Counterparts
Source: PLoS One. 2011 Sep 9;6(9):e24164. doi: 10.1371/journal.pone.0024164 (PMC3170333; doi:10.1371/journal.pone.0024164)
Supplement: Methods S1 — A detailed description of the materials and methods can be found in this supporting information file. (DOC) [file pone.0024164.s001.doc]

#### **Supplemental Materials and Methods S1**

#### Expanded Materials & Methods

#### **Isolation and culture of human mesenchymal stem cells (hMSCs)**

All human-derived tissues were collected based on individual written (parental) informed consent, after approval by the Medical Ethics committee of the Leiden University Medical Center (LUMC), where all investigations were performed. The investigation conforms with the principles outlined in the Declaration of Helsinki.

## Derivation of MSCs from human embryonic stem cells (hESCs)

MSCs were derived from undifferentiated hESC colonies (hES3 subclones) as previously described [1,2]. Briefly, the undifferentiated hESC colonies were removed from their mouse embryonic fibroblast (mEF) feeder layer and propagated in gelatin-coated culture dishes in standard MSC culture medium (Dulbecco's modified Eagle's medium [Invitrogen, Breda, The Netherlands] containing 10% fetal bovine serum [FBS; Invitrogen], penicillin [100 U/mL] and streptomycin [100 µg/mL]; hereinafter referred to as MSC-CM) at 37ºC in a humidified 5% CO_2_ incubator. After 2-3 days of culture, a portion of the cells at the periphery of the hESC colonies differentiated toward spindle-shaped fibroblast-like cells. Next, the undifferentiated portions of the hESC colonies were removed by physical scraping and suctioning. Consecutive enzymatic passaging as single cell suspensions led to a reproducible derivation of morphologically homogeneous fibroblast-like cells from cultures of pluripotent undifferentiated hESCs within 2-3 passages (n=5 different isolations). Medium was replaced twice a week until the primary cultures were 60-80% confluent, after which the so-called hESC-MSCs were amplified by serial passage using a buffered 0.05% trypsin-0.02% ethylenediaminetetraacetic acid/EDTA solution (TE; BioWhittaker, Vervier, Belgium) for cell detachment.

*Fetal hMSC isolation and culture*

Human fetal tissues (gestational age between 17-22 weeks) were collected through legal interventions by the Department of Obstetrics. Fetal umbilical cords (UCs) and amniotic membranes were washed twice with phosphate-buffered saline (PBS) and were finely minced into 1-2 mm fragments using scissors and scalpels. Cells were released by treatment with 0.1% collagenase type I (Worthington, Lakewood, NJ, USA) for 3 h. Thereafter, 10 mL MSC-CM was added. The cell suspension was transferred to a 25-cm^2^ culture flask (Becton Dickinson, Franklin Lakes, NJ, USA) and incubated for 3-4 days at 37ºC in a humidified 5% CO_2_ atmosphere to allow the cells to adhere. Fetal UC and amniotic membrane (amniotic) hMSCs were subcultured as described in the previous section. Single cell suspensions of fetal bone marrow (BM) were obtained by punching fetal *femora* and *tibiae* with a 23-gauge needle and flushing them with culture medium. The cell suspension was centrifuged at 330 *g* for 10 min after which the same culture methods were applied as for the other fetal hMSCs. To obtain human fibroblasts, sections of human fetal skin (5×5 mm) were transferred to 25-cm^2^ culture flasks containing 5 ml MSC-CM and maintained in a humidified 5% CO_2_ incubator at 37°C. Fetal human skin fibroblasts (hSFBs) were allowed to migrate from the skin sections for 7 days. Thereafter, the skin sections were removed and the remaining hSFBs were cultured using standard procedures.

## Adult MSC isolation and culture

Adult hMSCs were purified from leftover BM samples derived from adult donors undergoing orthopedic surgery (n=8 donors, mean donor age 72±2.4 yrs). Briefly, the mononuclear cell fraction of the BM was isolated by Ficoll density gradient centrifugation. Twenty-four hours after seeding of the BM mononuclear cell fraction in 75-cm^2^ culture flasks (Becton Dickinson), the non-adherent cells were removed and the remaining hMSCs were expanded by serial passage using standard methods.

Adult adipose tissue (adipose) hMSCs were derived from subcutaneous adbdominal fat tissue (n= 10 donors, mean donor age 39.6±1.1 yrs). Tissue samples were washed twice with PBS containing penicillin (100 U/mL) and streptomycin (100 µg/mL). For tissue disruption 0.1% collagenase type I solution was added and tissue samples were finely minced. Next, samples were incubated at 37°C in a humidified 5% CO_2_ incubator for 1 h. Collagenase type I activity was quenched by adding excess MSC-CM. Samples were then centrifuged at 330 *g* for 10 min. After centrifugation the layer of primary adipocytes could be removed and the collagenase type I-containing solution was aspirated. The cell pellet was resuspended in MSC-CM, filtered through a 70 μm cell strainer (Becton Dickinson) and the cells were once again collected by centrifugation. This step was repeated twice, after which the cell pellet was resuspended in 5 ml culture medium. The resulting cell suspension was transferred to a 25-cm^2^ culture flask and these adult adipose tissue (adipose) hMSCs were propagated as all the other hMSCs.

Isolation and culture of neonatal rat (nr) cardiomyocytes (CMCs) and cardiac fibroblasts (CFBs)

All animal experiments were approved by the Animal Experiments Committee of the LUMC and conform to the Guide for the Care and Use of Laboratory Animals, as stated by the US National Institutes of Health (permit numbers: 09012 and 10236) [3].

nrCMCs and nrCFBs were dissociated from ventricles of 2-day old male Wistar rats, separated from each other by differential plating and maintained in nrCMC culture medium containing 5% horse serum (Invitrogen), penicillin (100 U/mL; BioWhittaker) and streptomycin (100 µg/mL; BioWhittaker), as previously described [4]. nrCFBs were cultured in MSC-CM and passaged at least three times before they were used in co-culture experiments.

Five hundred thousand or one million nrCMCs were plated on collagen type I -coated (Sigma-Aldrich) glass coverslips in 6-well culture dishes and incubated in a humidified incubator at 37 C and 5% CO_2_. Proliferation of residual nrCFBs in nrCMC cultures was inhibited by incubation of the cells with 100 μmol/L 5-bromo-2-deoxyuridine (Sigma-Aldrich) during the first 24 h after culture initiation.

For optical mapping experiments, 8×10^4^ nrCMCs were plated on fibronectin -coated (Sigma-Aldrich) glass coverslips in 24-well culture dishes. As the conduction velocity (CV) through monolayers of nrCMCs is inversely related to their CFB content, prior to their use in co-incubation experiments with hMSCs, the nrCMC cultures were treated for 2 h with 10 μg/mL mitomycin-C (Sigma-Aldrich) to stop proliferation of residual nrCFBs present in these cultures [12].

**Characterization of hMSCs**

Flow cytometry

Analysis of surface marker expression was carried out by flow cytometry. hMSCs (passage≥3) were detached using TE, resuspended in PBS containing 1% bovine serum albumin fraction V (BSA; Sigma-Aldrich) and divided in aliquots of 2×10^5^ cells. Cells were then incubated for 30 min at 4°C with fluorescein isothiocyanate-, phycoerythrin- or allophycocyanin-conjugated antibodies directed against human CD105 (Ancell, Bayport, MN, USA), CD90, CD73, CD45, CD34, CD31, CD24 and stage-specific embryonic antigen-4 (SSEA-4) (all from Becton Dickinson). Labeled cells were washed three times with PBS containing 1% BSA and analyzed using an LSR II three-laser, 12-color, flow cytometer (Becton Dickinson). Isotype-matched control antibodies (Becton Dickinson) were used to determine background fluorescence. At least 10^4^ cells per sample were acquired and data were processed using FACSDiva software (Becton Dickinson).

Adipogenic and osteogenic differentiation of hMSCs

The hMSCs were characterized by established differentiation assays [5]. Briefly, 5x10^3^ hMSCs per well were plated in a 12-well culture plate and exposed to adipogenic or osteogenic differentiation medium. Adipogenic differentiation medium consisted of MEM-plus (i.e. α-minimum essential medium [Invitrogen] containing 15% FBS, 100 U/L penicillin and 100 µg/mL streptomycin) supplemented with insulin, dexamethason, indomethacin and 3-isobutyl-1-methylxanthine (all from Sigma-Aldrich) to final concentrations of 5 µg/mL, 1 µM, 50 µM and 0.5 µM, respectively, and was refreshed every 3-4 days for a period of 3 weeks. Lipid accumulation was assessed by Oil Red O (Sigma-Aldrich) staining of the cultures (15 mg of Oil Red O/mL of 60% isopropanol) and light microscopy. Osteogenic differentiation medium consisted of MEM-plus containing 10 mM β-glycerophosphate, 50 µg/mL ascorbic acid and 10 nM dexamethason (all from Sigma-Aldrich) and was refreshed every 3-4 days for a period of 3 weeks. Afterwards, the cells were washed with PBS and calcium deposits were visualized by staining of the cells for 5 min with 2% Alizarine Red S (Sigma-Aldrich) in 0.5% NH_4_OH (pH 5.5).

*Immunocytological characterization of hESC-MSCs*

hESC colonies were originally grown on a feeder layer of mEFs. To verify the human origin of the fibroblast-like cells derived from the hESC colonies, the cells were incubated with a monoclonal antibody (MAb) specific for human lamin A/C (clone 636; Vector laboratories, Burlingame, CA, USA) at a dilution of 1:200, as previously described [6]. Binding of the primary antibody to its target antigen was visualized using Alexa 568-linked donkey anti-mouse IgG secondary antibodies (Invitrogen; dilution 1:200). Murine MSCs (mMSCs) were not labeled with the human lamin A/C-binding MAb, confirming its species specificity.

In addition, hESC-MSCs were stained with antibodies directed against the hESC marker SSEA-4 (MAb MC813; Santa Cruz Biotechnologies, Santa Cruz, CA, USA) or against the pluripotency-associated transcription factors Oct-3/4 (MAb N-19; Santa Cruz) and Nanog (goat polyclonal antibody [PAb]; R&D Systems, Minneapolis, MN, USA). Each of these primary antibodies was applied at a dilution of 1:100. As secondary antibodies we used Alexa 568-conjugated donkey anti-mouse IgG or donkey anti-goat IgG (both from Invitrogen) at a dilution of 1:200. hESC colonies from which the hESC-MSCs were derived, were used as a positive control in these stainings. Lastly, hESC-MSCs were stained with fluorescein isothiocyanate- and phycoerythrin- conjugated antibodies directed against the mesenchymal stem cell markers CD90, CD73 and CD105 at a dilution of 1:200.

Growth kinetics

Growth kinetics of the hMSCs was analyzed by calculating population doublings (PDs). Each type of hMSC was plated in triplicate at a concentration of 2x10^3^ hMSCs per cm^2^ in 25-cm^2^ culture flasks (n=3 isolations for each type of hMSC). After every 5 days of culture at 37°C in 95% humidified air-5% CO_2_, the cells were trypsinized and resuspended in 5 mL. Subsequently, a part of each cell suspension was subjected to Trypan Blue staining to determine the viable cell concentration using a hemocytometer. This information was used to initiate the next cell passage in a new culture flask and to determine the number of PDs during the previous culture period.

*Relative telomere length using quantitative real-time polymerase chain reaction (qRT-PCR)*

Genomic DNA from experimental (n≥6 samples of ESC-derived, fetal or adult MSCs cultured for the same period of time) and reference samples was obtained using DNAzol (Invitrogen) according to the recommendations of the manufacturer. Relative telomere lengths were measured by SYBR Green-based (QuantiTect SYBR Green PCR kit; Qiagen, Valencia, CA, USA) qRT-PCR amplification of telomere repeats (T) and single-copy gene *36B4* (S) in a LightCycler 480 Real-Time PCR System (Roche, Foster City, CA, USA). The *36B4* gene was analyzed to normalize for differences in DNA amount between samples. The primers, primer concentrations and thermal cycling profiles were identical to those of Cawthon *et al* [7]. T and S standard curves were generated using serial dilutions (100 to 20 ng) of the DNA from the reference sample. The telomere- and *36B4*-specific qPCRs were carried out in separate plates and a standard curve was produced in each run to allow relative quantification between samples (50 ng per sample). The T/S ratio of one sample relative to that of another corresponds to the relative telomere lengths of their DNA. Since the amount of PCR product approximately doubles during each amplification cycle, the T/S ratio is approximately [2^Ct(telomeres)^/ 2^Ct(36B4)^]^-1^ = 2^-ΔCt^. The relative T/S ratio is 2^-(ΔCt1-ΔCt2)^ = 2^-ΔΔCt^.

**Cardiac differentiation potential of hMSCs derived from different sources**

To facilitate the identification of hMSCs in co-cultures with nrCMCs or nrCFBs, these cells were transduced with *enhanced green fluorescent protein* (eGFP) using the vesicular stomatitis virus G protein-pseudotyped self-inactivating human immunodeficiency virus type 1 (HIV-1) vector CMVPRES [8], essentially as described by van Tuyn *et al* [9]*.* Before being used in co-culture experiments, the *eGFP*-transduced hMSCs were subcultured for several passages to avoid undesired secondary transductions of nrCMCs or nrCFBs by infectious HIV-1 particles carried over by the hMSCs. Cardiomyogenic differentiation was studied in co-cultures of 5x10^4^ eGFP-labeled hMSCs and 5x10^5^ nrCMCs or 5x10^5^ nrCFBs. As a control group, 5x10^4^ eGFP-labeled fetal hSFBs were co-incubated with 5x10^5^ nrCMCs. The eGFP-labeled hMSCs and fetal hSFBs were added to the nrCMCs two days after they had been isolated and put into culture. All experiments described below were conducted using hMSCs from passage 3-6.

Human-specific immunocytochemical analysis of cardiomyogenic differentiation potential

Co-cultures of 5x10^5^ nrCMCs and 5x10^4^ eGFP-labeled hMSCs or eGFP-labeled fetal hSFBs and co-cultures of 5x10^5^ nrCFBs with 5x10^4^ eGFP-labeled hMSCs were stained with a MAb recognizing the sarcomeric protein α-actinin (clone EA53; Sigma-Aldrich; dilution 1:400) on day 10 after culture initiation, as previously described [6]. The primary antibody was visualized using Alexa 568-coupled donkey anti-mouse IgG secondary antibodies at a dilution of 1:200. The human lamin A/C-specific MAb mentioned above was used to detect hMSCs in the co-cultures. Lamin A/C staining was visualized with Qdot 655-streptavidin conjugates (Invitrogen) after incubation of the cells with biotinylated goat anti-mouse IgG2b secondary antibodies (Santa Cruz). Nuclei were stained using a 10 µg/mL solution of Hoechst 33342 (Invitrogen) in PBS containing 1% FBS. The percentage of eGFP-labeled cells showing positive staining for α-actinin was determined by analyzing at least 3 cultures (100 cells per culture, at 100x magnification) of at least 4 hMSC isolations per type of hMSC). The presence of well-organized sarcomeres in eGFP-labeled hMSCs was assessed by comparing the α-actinin staining pattern in these cells with that of the native nrCMCs in each culture.

A fluorescence microscope equipped with a digital camera (Nikon Eclipse, Nikon Europe, Badhoevedorp, The Netherlands) and dedicated software (Image-Pro Plus, Version 4.1.0.0, Media Cybernetics, Silver Spring, MD, USA) were used to analyze data. All co-cultures were treated equally using the same antibody dilutions and exposure times.

*Electrophysiological measurements in pharmacologically uncoupled hMSCs in co-culture with nrCMCs*

Whole-cell patch-clamp measurements were performed in co-cultures of 5x10^4^ eGFP-labeled fetal (amniotic) or adult (adipose) hMSCs and 5x10^5^ nrCMCs, plated on collagen-coated glass coverslips, at day 10 of culture. To perform single-cell measurements from eGFP-labeled cells in a field of beating nrCMCs, cells were pharmacologically uncoupled by incubation with 180 µmol/L of 2-aminoethoxydiphenyl borate (2-APB) (Tocris, Ballwin, MO, USA) for 15 min [6]. This agent blocks gap junctional intercellular coupling by Cx40, Cx43, and Cx45 [10,11]. Whole-cell current-clamp recordings were performed at 25°C using a L/M-PC patch-clamp amplifier (3 kHz filtering) (List-Medical, Darmstadt, Germany). Pipette solution contained (in mmol/L) 10 Na_2_ATP, 115 KCl, 1 MgCl_2_, 5 EGTA, 10 HEPES/KOH (pH 7.4). Tip resistance was 2.0 - 2.5 MΩ, and seal resistance >1 GΩ. The bath solution contained (in mmol/L) 137 NaCl, 4 KCl, 1.8 CaCl_2_, 1 MgCl_2_, 10 HEPES (pH 7.4). For data acquisition and analysis pClamp/Clampex8 software (Axon Instruments, Molecular Devices, Sunnyvale, CA, USA) was used. Current-clamp recording were performed in eGFP-labeled cells which were adjacent to 2-4 nrCMCs, and from these cells the data were analyzed and compared between the two different groups.

Human-specific quantitative reverse transcription-PCR (qRT-PCR) to detect mRNAs associated with pluripotency and cardiac differentiation

Total cellular RNA was extracted from monocultures of hMSCs (n≥4 samples from each type of hMSC) and from co-cultures consisting of 10^5^ hMSCs and 10^6^ nrCMCs using the RNeasy Mini kit (Qiagen). Oligo (dT)-primed reverse transcription was performed on 2 µg of total cellular RNA and the resultant cDNA was used for PCR amplification using SYBR Green. To detect changes in cardiac and pluripotency gene expression levels, the following human-specific primers: gap junction protein, alpha 1 (Cx43/GJA1; QT00012684), vascular endothelial growth factor A (VEGF/VEGFA; QT01682072), GATA-binding protein 4 (GATA-4/GATA4; QT00031997), Nanog homeobox (Nanog/NANOG; QT01844808), octamer-binding protein 3/4 (Oct-3/4/POU5F1; QT00210840), NK2 transcription factor related, locus 5 (Drosophila) (Nkx2.5/NKX2-5; QT00010619), v-kit Hardy-Zuckerman 4 feline sarcoma viral oncogene homolog (c-kit/KIT; QT01844549), natriuretic peptide precursor A (ANP/NPPA; QT00203322), myosin, light chain 2, regulatory, cardiac, slow (MLC2v/MYL2; QT00012999), ISL LIM homeobox 1 (Islet-1/ISL1; QT00000294), troponin I type 3 (cardiac) (cTnI/TNNI3; QT00084917) (all with an annealing temperature of 55°C; all from Qiagen) and myosin heavy chain 7, cardiac muscle, beta (β-MCH/MYH7; forward primer: 5’-TGTGTCACCGTCAACCCTTA-3’, reverse primer: 5’-TGGCTGCAATAACAGCAAAG-3’; annealing temperature 63°C; Invitrogen). The expression of the genes of interest was normalized to that of the housekeeping gene *glyceraldehyde-3-phosphate dehydrogenase* (GAPDH, forward primer: 5´-GAAGGTGAAGGTCGGAGTC-3´, reverse primer: 5´-GAAGATGGTGATGGGATTTC-3´; annealing temperature 60°C; Invitrogen). Agarose gel electrophoresis was used to ensure that each primer pair yielded a single PCR product of the expected size. PCR primers were checked for human specificity with the aid of appropriate positive human right atrium and hESC control and negative nrCMC control samples, while rat-specific primers were used to detect expression of the cardiac genes in the nrCMC control samples. Data were analyzed using the ΔCt method.

Optical mapping to determine CV in co-cultures between nrCMCs and different types of hMSCs

Action potential propagation was investigated on a whole-culture scale in wells of a 24-well plate by optically mapping using the voltage-sensitive dye di-4-ANEPPS (Invitrogen). The measurements were performed 10 days after seeding of either 8x10^5^ nrCMCs (nrCMC monoculture) or 8x10^5^ nrCMCs plus 8x10^4^ nrCFBs or 8x10^4^ hMSCs (nrCMC/nrCFB or nrCMC/hMCS co-cultures) per well (n≥15 cultures per cell type or combination of cell types). Co-cultures were loaded with 16 μmol/L di-4-ANEPPS for 30 minutes. After which medium was refreshed and the co-cultures were mapped using the Ultima-L optical mapping setup (SciMedia, Costa Mesa, CA, USA). Throughout mapping experiments, cultures were kept at 37ºC. Optical signal recordings were analyzed using Brain Vision Analyze 0909 (Brainvision Inc, Tokyo, Japan). For more details regarding the optical mapping protocol see Askar *et al* [12]. The CV of all (co-)cultures was determined in a blinded manner.

**In vitro angiogenesis assays**

hMSCs of different origin (n≥5 isolations for each type of hMSC) were plated on Matrigel (Becton Dickinson) to determine their ability to form capillary-like structures. Ninety microliters of gel matrix solution was applied to each well of a 24-well plate on top of a glass coverslip and the plate was incubated for 1 h at 37°C. After trypsinization, 1.5x10^4^ cells were suspended in 1 mL of Endothelial Growth Medium-2 (Cambrex IEP, Wiesbaden, Germany) containing 100 ng/mL recombinant human VEGF-A_165_ (R&D Systems), plated onto the basement membrane matrix and incubated for up to 24 h at 37°C in 95% humidified air-5% CO_2_. Formation of capillary-like structures was checked every hour. Maximum time of incubation was determined for each type of hMSC. Following culture on the basement membrane matrix, cells were fixed and stained with antibodies specific for smooth muscle myosin heavy chain (smMHC; MAb hSM-V; Sigma-Aldrich, dilution 1:100) and platelet/endothelial cell adhesion molecule-1 (PECAM-1; rabbit PAb M20; Santa Cruz, dilution 1:200). The primary antibodies were visualized with Alexa 568-coupled donkey anti-mouse IgG and Alexa 488-conjugated donkey anti-rabbit IgG (Invitrogen), respectively. All cultures were treated equally using the same antibody dilutions and exposure times, which were based on titration of the antibodies using appropriate positive and negative controls.

**Determination of Cx43 expression**

Cx43 protein levels and gene expression were detected in monocultures of hESC-MSCs, fetal amniotic hMSCs and adult adipose hMSCs, but also in co-cultures of these cells with nrCMCs or nrCFBs using immunocytology and qRT-PCR, as described earlier. The Cx43-specific rabbit PAb (C6219; Sigma Aldrich, dilution 1:200) was visualized with Alexa 488-conjugated donkey anti-rabbit IgG. Cx43 expression was determined for at least 5 different isolations of each type of hMSCs under the different conditions (100 cells per culture and at least 3 cultures per isolation were analyzed). All cultures were treated equally using the same antibody dilutions and exposure times, which were based on titration of the antibodies using appropriate positive and negative controls.

Western blot analysis was used to quantify Cx43 levels in cultures of hMSCs. Homogenates were made from at least 5 different isolations of hMSCs per source. After determining the protein concentration in each sample using the BCA Protein Assay Reagent (Pierce Biotechnology, Rockford, IL, USA), equal amounts of protein were size-fractionated in a 12% NuPage Tris-Acetate gel (Invitrogen) and transferred to a Hybond-P PVDF membrane (GE Healthcare, Waukesha, WI, USA). This membrane was incubated for 1 h with the PAb directed against Cx43 followed by incubation with horse radish peroxidase (HRP)-conjugated goat anti-rabbit secondary antibody (Santa Cruz). To check for equal protein loading, a mouse MAb recognizing the housekeeping protein GAPDH (Chemicon International, Temecula, CA, USA) was used, which was detected by an HRP-conjugated goat anti-mouse secondary antibody (Santa Cruz). Chemiluminescence was induced with the aid of the ECL Advance Western Blotting Detection Kit and caught on Hyperfilm ECL (both from GE Healthcare).

References

1. Trivedi P, Hematti P (2008) Derivation and immunological characterization of mesenchymal stromal cells from human embryonic stem cells. Exp Hematol 36: 350-359.

2. Karlsson C, Emanuelsson K, Wessberg F, Kajic K, Axell MZ et al. (2009) Human embryonic stem cell-derived mesenchymal progenitors-Potential in regenerative medicine. Stem Cell Res.

3. National Institutes of Health (2002) Guide for the Care and Use of Laboratory Animals.

4. Pijnappels DA, Schalij MJ, van Tuyn J, Ypey DL, de Vries AA et al. (2006) Progressive increase in conduction velocity across human mesenchymal stem cells is mediated by enhanced electrical coupling. Cardiovasc Res 72: 282-291.

5. Pittenger MF, Mackay AM, Beck SC, Jaiswal RK, Douglas R et al. (1999) Multilineage potential of adult human mesenchymal stem cells. Science 284: 143-147.

6. Pijnappels DA, Schalij MJ, Ramkisoensing AA, van Tuyn J, de Vries AA et al. (2008) Forced alignment of mesenchymal stem cells undergoing cardiomyogenic differentiation affects functional integration with cardiomyocyte cultures. Circ Res 103: 167-176.

7. Cawthon RM (2002) Telomere measurement by quantitative PCR. Nucleic Acids Res 30: e47.

8. Seppen J, Rijnberg M, Cooreman MP, Oude Elferink RP (2002) Lentiviral vectors for efficient transduction of isolated primary quiescent hepatocytes. J Hepatol 36: 459-465.

9. van Tuyn J, Pijnappels DA, de Vries AA, de V, I, van der Velde-van Dijke et al. (2007) Fibroblasts from human postmyocardial infarction scars acquire properties of cardiomyocytes after transduction with a recombinant myocardin gene. FASEB J 21: 3369-3379.

10. Bai D, del CC, Srinivas M, Spray DC (2006) Block of specific gap junction channel subtypes by 2-aminoethoxydiphenyl borate (2-APB). J Pharmacol Exp Ther 319: 1452-1458.

11. Harks EG, Camina JP, Peters PH, Ypey DL, Scheenen WJ et al. (2003) Besides affecting intracellular calcium signaling, 2-APB reversibly blocks gap junctional coupling in confluent monolayers, thereby allowing measurement of single-cell membrane currents in undissociated cells. FASEB J 17: 941-943.

12. Askar SFA, Ramkisoensing AA, Schalij MJ, Bingen BO, van der Laarse A, Atsma DE, Ypey DL, Pijnappels DA (2011) Antiproliferative Treatment of Endogenous Myofibroblasts Prevents the Occurrence of Spontaneous Reentrant Tachyarrhythmias in Rat Myocardial Cultures. Cardiovasc Res 90: 295-304.
