# Supplementary figures and images for: Human Embryonic and Fetal Mesenchymal Stem Cells Differentiate toward Three Different Cardiac Lineages in Contrast to Their Adult Counterparts
Source: PLoS One. 2011 Sep 9;6(9):e24164. doi: 10.1371/journal.pone.0024164 (PMC3170333; doi:10.1371/journal.pone.0024164)

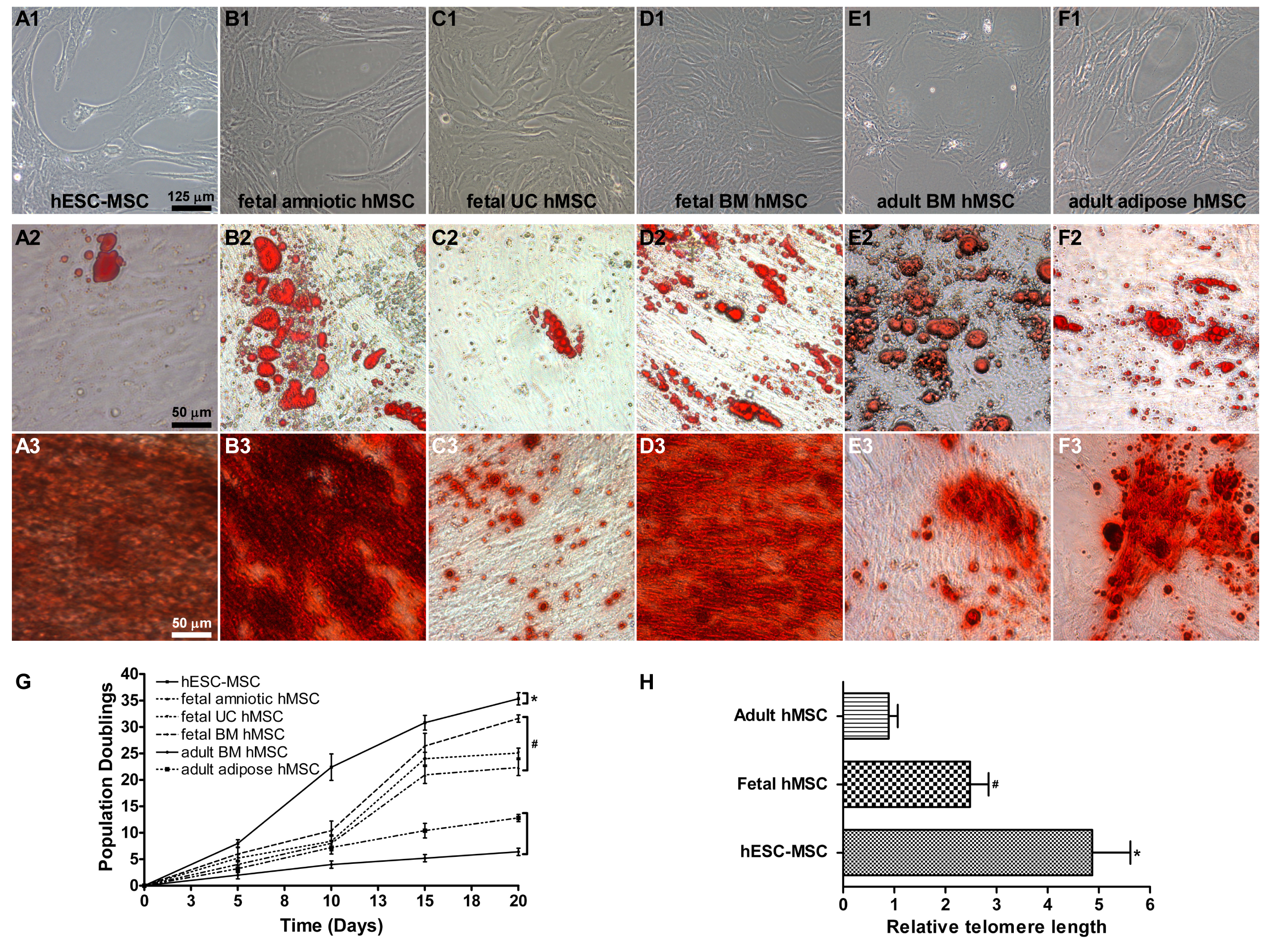

Supplement: Figure S1 — Cellular characteristics of hMSCs. (A1–F1) Bright field images of cultured hMSCs displaying a spindle-shaped morphology. (A2–F2) Presence of oil red O-stained fat vacuoles after adipogenic differentiation. (A3–F3) Calcium depositions after osteogenic differentiation was visualized by alizarine red S staining. (A) hESC-MSC; (B) fetal amniotic hMSC; (C) fetal UC hMSC; (D) fetal BM hMSC; (E) adult BM hMSC; (F) adult adipose hMSC. (G) Growth kinetics of the different types of hMSCs estimated by cumulative population doublings over 20 days (* P<0.001 vs fetal hMSCs and adult hMSCs, # P<0.001 vs adult hMSCs). (H) Mean relative telomere lengths of hESC-MSCs, all fetal hMSC types and both adult hMSC types (* P<0.05 vs fetal hMSCs and adult hMSCs, # P<0.05 vs adult hMSCs). (TIF) [file pone.0024164.s002.tif]

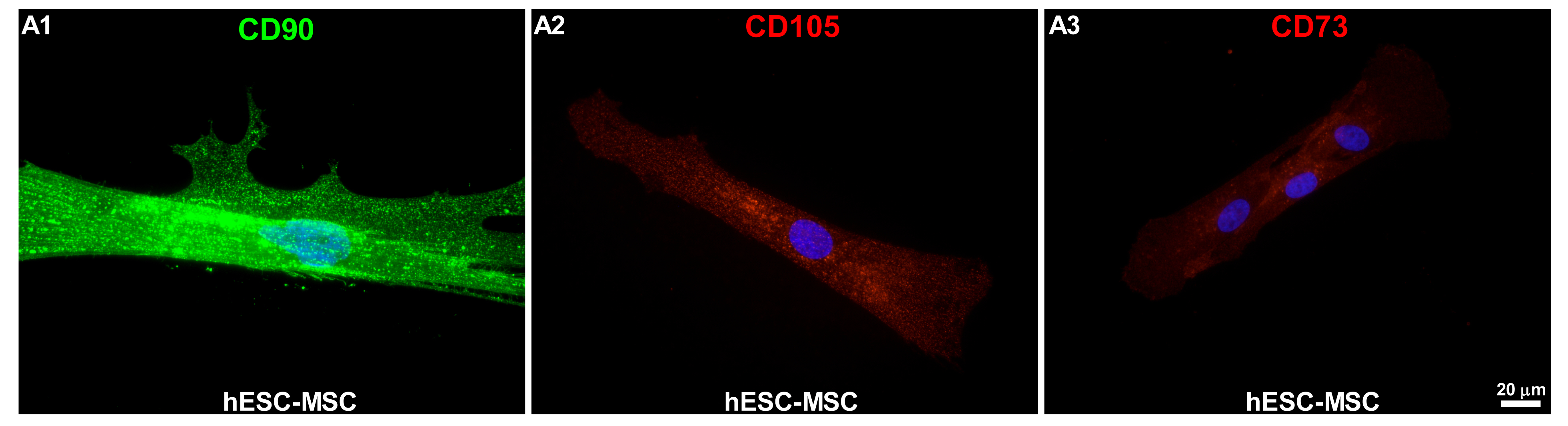

Supplement: Figure S2 — Immunocytological characterization of hESC-MSCs for MSC surface markers. Immunostaining of hESC-MSCs for CD90, CD105 and CD73 (A1–A3) showed that these cells were positive for these established MSC surface markers. (TIF) [file pone.0024164.s003.tif]
