# Supplementary material for: Human Embryonic and Fetal Mesenchymal Stem Cells Differentiate toward Three Different Cardiac Lineages in Contrast to Their Adult Counterparts
Source: PLoS One. 2011 Sep 9;6(9):e24164. doi: 10.1371/journal.pone.0024164 (PMC3170333; doi:10.1371/journal.pone.0024164)
Supplement: Table S1 — Analysis of surface marker expression. All hMSC types were positive for the established MSC surface markers CD105, CD90 and CD73. They were negative for the hematopoietic, endothelial and embryonic stem cell markers CD45 and CD34, CD31 and SSEA-4, respectively. The hESC-MSCs were also negative for CD24, a protein present on the surface of hESCs. Mean percentages ± standard deviations are given; n = 6 for each group. NT is not tested. (DOC) [file pone.0024164.s004.doc]

|  | **hESC-MSC** | **Fetal amniotic hMSC** | **Fetal BM hMSC** | **Fetal UC hMSC** | **Adult BM hMSC** | **Adult adipose hMSC** |
| --- | --- | --- | --- | --- | --- | --- |
| **CD24** | 0.30±0.7 | NT | NT | NT | NT | NT |
| **CD31** | 0.0±0.0 | 0.0±0.0 | 0.0±0.0 | 0.0±0.0 | 0.0±0.0 | 0.0±0.0 |
| **CD34** | 0.13±0.1 | 0.0±0.0 | 0.13±0.1 | 0.0±0.0 | 0.17±0.1 | 0.06±0.0 |
| **CD45** | 0.20±0.4 | 0.07±0.0 | 0.10±0.1 | 0.18±0.1 | 0.20±0.1 | 0.6±0.5 |
| **CD73** | 97.6±1.9 | 95.4±0.8 | 97.3±1.3 | 97.7±1.1 | 96.6±1.4 | 96.4±0.3 |
| **CD90** | 96.0±1.7 | 98.2±1.3 | 96.3±1.5 | 96.3±3.1 | 98.2±0.9 | 94.6±1.8 |
| **CD105** | 94.0±0.9 | 95±0.9 | 96.6±0.2 | 97.4±0.6 | 96.0±2.8 | 96.0±0.2 |
| **SSEA-4** | 0.0±0.0 | 0.0±0.0 | 0.0±0.0 | 0.0±0.0 | 0.0±0.0 | 0.0±0.0 |
