# Supplementary material for: Human Embryonic and Fetal Mesenchymal Stem Cells Differentiate toward Three Different Cardiac Lineages in Contrast to Their Adult Counterparts
Source: PLoS One. 2011 Sep 9;6(9):e24164. doi: 10.1371/journal.pone.0024164 (PMC3170333; doi:10.1371/journal.pone.0024164)
Supplement: Table S2 — qRT-PCR analysis to detect mRNAs associated with cardiac differentiation. Indicated is the fold change in the expression of cardiac genes in hMSCs cultured alone or together with nrCMCs. # P<0.05 vs hMSC monoculture; * P<0.01 vs hMSC monoculture; †P<0.001 vs hMSC monoculture; ND is not detected. (DOC) [file pone.0024164.s005.doc]

|  | **Nkx2.5** | **GATA-4** | **ANP** | **MLC2v** | **Cx43** | **VEGF** | **Islet-1** | **c-kit** |
| --- | --- | --- | --- | --- | --- | --- | --- | --- |
| **hESC-MSC** | 6.15±1.2* | 5.77±0.6# | 2.97±0.1† | 12.0±0.6† | 9.13±0.9# | 1.18±0.3 | 0.43±0.1* | 0.53±0.2# |
| **Fetal amniotic hMSC** | 7.26±0.4# | ND | 1.56±0.1* | ND | 3.94±0.5* | 3.85±0.3* | 0.35±0.1# | 0.75±0.2 |
| **Fetal UC hMSC** | ND | 5.79±1.4# | 1.04±0.1 | ND | 2.72±0.2# | 2.32±0.1† | 0.50±0.1† | 0.36±0.1* |
| **Fetal BM hMSC** | ND | ND | 3.73±0.1† | ND | 1.97±0.3# | 0.93±0.1 | 6.13±1.9 | 6.13±1.9† |
| **Adult BM hMSC** | ND | ND | 2.07±0.4# | ND | 1.03±0.3 | 1.44±0.8* | ND | 0.84±0.3 |
| **Adult adipose hMSC** | ND | ND | 5.18±1.5# | ND | 1.22±0.2 | 11.7±1.8* | ND | 5.71±1.0# |
